# Epitheliod Leiomyoma of the Bladder: An Unusual Case of Irritative and Obstructive Voiding Symptoms

**DOI:** 10.1155/2012/759150

**Published:** 2012-05-31

**Authors:** Ali Kaviani, Abdollah Razi, Hooman Mokhtarpour, Mohammad Mohsen Mazloomfard, Aida Moeini, Hooman Bahrami-Motlagh

**Affiliations:** ^1^Department of Urology, Tajrish Hospital, Shaheed Beheshti University of Medical Science, Tehran 19395-4763, Iran; ^2^Department of Gynecology, Tajrish Hospital, Shaheed Beheshti University of Medical Science, Tehran 19395-4763, Iran; ^3^Department of Radiology, Tajrish Hospital, Shaheed Beheshti University of Medical Science, Tehran 19395-4763, Iran

## Abstract

Epitheloid leiomyoma is a very rare subtype of benign mesothelial tumors of the bladder. A 46-year-old female patient presented to our hospital with prolonged dysuria, frequency, and recurrent urinary tract infections. Bimanual examination revealed a mobile, round mass in bladder. There was a round hyperdense intravesical mass near bladder neck in computed tomography (CT) scan that was compatible with her magnetic resonance imaging (MRI). A well defined 3 × 4 centimeter mass was seen in superolateral part of bladder neck during cystoscopy. The patient underwent partial cystectomy and histopathologic findings confirmed the diagnosis of epithelioid leiomyoma. The patient's followup was uneventful in a period of 2 years. Size and anatomic location of this tumor were major factors that affect on treatment.

## 1. Introduction

Benign mesothelial tumors are rare cause of bladder neoplasms [1, 2]. Leiomyoma as a benign mesothelial tumor consists of four different subtypes and its epitheloid subtype is a very rare ones. This benign disorder results in irritative and obstructive urinary symptoms or hematuria [3–5]. We report a case of bladder epitheloid leiomyoma presenting with irritative symptoms and cystoscopic appearance of a well-defined mass in bladder which is a rare finding even in uterus [6].

## 2. Case Report

**Figure 1:**
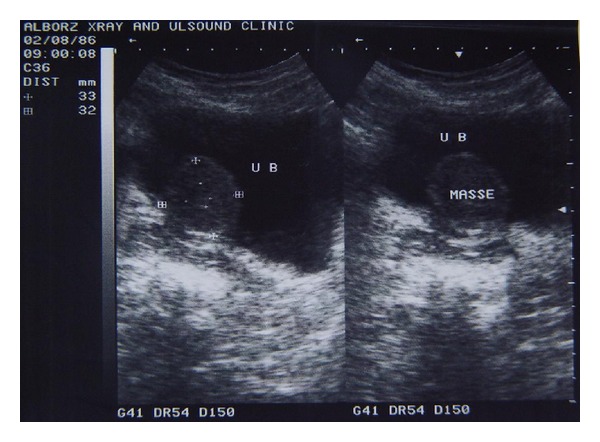
A heteroechoic round mass was revealed in ultrasonography.

**Figure 2:**
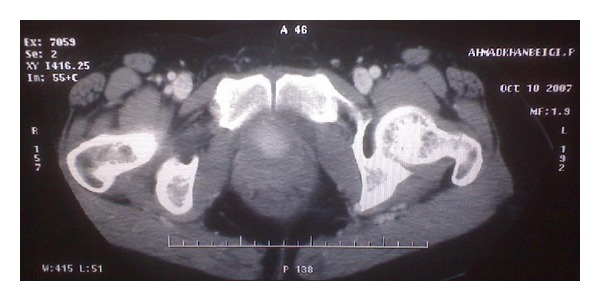
An intravesical mass near bladder neck with hyperdensity in CT scan of patient.

**Figure 3:**
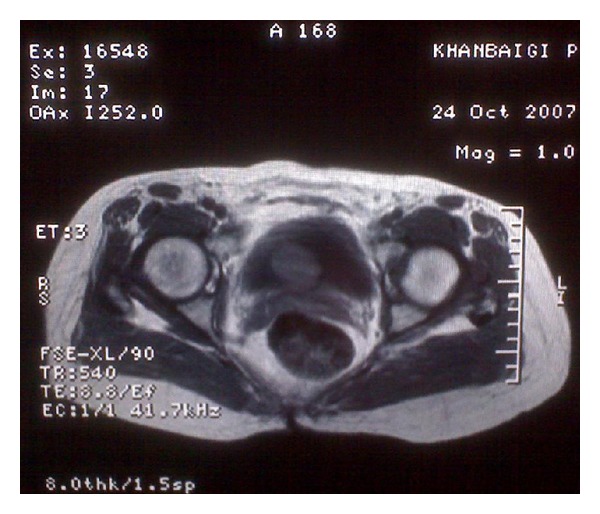
A well-defined 3 × 4 centimeter intramural mass was shown in MRI.

A 46-years-old woman presented to our hospital with complaining of dysuria, frequency, and recurrent urinary tract infections from one year ago. She had no hematuria, suprapubic pain, or family history of such problems. She had history of hysterectomy three years before this admission due to uterine leiomyoma. Physical examination revealed a mobile, round mass in bimanual examination. Abdominal ultrasonography revealed a round mass measuring 4 × 5 cm (Figure 1). There was no hydronephrosis in both kidneys. There was a round hyperdense intravesical mass near bladder neck in CT scan (Figure 2). The mass was intramural in MR imaging and showed intermediate signal intensity on T1-weighted images (Figure 3). A well defined 3 × 4 centimeter mass was seen in superolateral part of bladder neck during cystoscopy. There was no any involvement of ureteral orifices. Due to huge size of the mass and its proximity to ureteral orifice, trans-urethral bladder resection was not planned for patient.

She underwent exploratory laparotomy with low midline incision. The mass was identified in the posterior aspect of the bladder wall so partial cystectomy was performed for her. The pathology of mass was epithelioid leiomyoma. The postoperative period was uneventful, and the patient was discharged from hospital after 4 days.

The patient's followup with clinical examination, abdominopelvic CT scan, and cystoscopy was normal in period of 2 years. 

## 3. Discussion

Benign bladder tumors consist of myomas, fibromyomas, leiomyomas, rhabdomyomas, fibromas, angiomas, myxomas, and osteomas. The most common histologic type of benign bladder tumor is leiomyoma which does not have any predilection for gender or age [1]. In 1994, Goluboff et al. observed a higher incidence of leiomyoma in women of third to fifth decade of life with a mean size of 5.5 cm [1]. These lesions may be categorized as endovesical, extravesical, or intramural [7]. Endovesical masses have been mostly recognizable (63%); possibly due to its characteristic bulging into the bladder which induced irritative symptoms and forced the patient to seek medical treatment. The two other types of mass occur with a frequency of 30% and 7% for extravesical and intramural, respectively, [1]. Epitheloid leiomyoma is a rare subtype with uterus as a common site of involvement. Variations of leiomyoma in uterus include lipoleiomyomas, cellular leiomyomas, bizarre (symplastic) leiomyomas, and epithelioid leiomyomas [6]. Urinary symptoms were often observed in this tumor; for example, Goluboff et al. reviewed all reported cases of leiomyoma of the bladder in the English literature and indicated that obstructive symptoms were the most frequent (49%) presenting patient complaint [8]. On the other hand, Knoll et al. observed irritative symptoms as the most frequent presenting symptoms [9]. After a comprehensive history, a complete physical examination could be helpful, due to the fact that palpable lesion was encountered in 57% of women at bimanual examination.

In imaging such as intravenous urography (IVU) or cystourethrogram usually a smooth filling defect in the bladder was identified. Abdominal ultrasound may be helpful in differentiating cystic lesion from solid one [1, 9]. Fernandez and Dehesa investigated the various radiographic measures used in demonstrating these benign tumors to determine which method was the most fruitful for the physician preoperatively. They noted computed tomography (CT) scan to be beneficial for precisely locating the tumor but inadequate for identifying its relationship to the adjacent bladder mucosa or vaginal wall due to its fixed axial plane. They advocated transvaginal ultrasound with production of better definition of the mass. Leiomyomas of the bladder are demonstrated sonographically as solid smooth-walled lesions with many internal echoes and with an underlying homogeneous texture of medium echogenicity.

These tumors are easily visualized in cystoscopy that covered the normal bladder mucosa [10]. In histopathology, bladdrer leiomyoma may be clearly differentiated from leiomyosarcoma. Leiomyomas appear as whitish gray round to ovoid nodules with spiral appearance of smooth muscle fibers. They are firm and rubbery in consistency [8]. In contrast, leiomyosarcoma has little mitotic activity and usually has a large quantity of myxoid intracellular material with invasion to the muscularis propria. There are no reports of malignant degeneration of leiomyoma [8, 9].

The pathophysiology of leiomyoma of the bladder is unclear. However, four major theories have been proposed for this tumor: (1) inflammatory resection of the bladder wall to an infection of the bladder musculature; (2) metaplastic reaction around the perivascular walls from vascular inflammation of the bladder; (3) neoplasm controlled by hormonal influences; (4) dysontogenesis: the result of embryologic rest within the bladder wall developing into a smooth muscle tumor. Despite of mysterious cause of it, the diagnostic examination and surgical management are easy [1, 8].

The treatment of this rare condition is primarily determined by its size and anatomic location [1]. In Goluboff's review, 62% were treated by open resection, whereas 30% were removed transurethral [8]. In patients with large endovesical tumors, extravascular tumor, or intramural lesions open resection is a good option and does not require a second procedure. Patient becomes asymptomatic after surgical excision because of low reoperation rate, absence of recurrence, and excellent prognosis of leiomyoma [1, 8, 9].

In our patient, irritative voiding symptoms were predominant and intramural lesion was diagnosed by imaging modalities. The normal bladder mucosa over the mass confirmed intramural situation of lesion. A straightforward partial cystectomy was performed for her.

## References

[1] Soloway D, Simon MA, Milikowski C, Soloway MS (1998). Epithelioid leiomyoma of the bladder: an unusual cause of voiding symptoms. *Urology*.

[2] Bai SW, Jung HJ, Jeon MJ, Jung DJ, Kim SK, Kim JW (2007). Leiomyomas of the female urethra and bladder: a report of five cases and review of the literature. *International Urogynecology Journal and Pelvic Floor Dysfunction*.

[3] Campbell EW, Gislason GJ (1953). Benign mesothelial tumors of the urinary bladder: review of literature. *The Journal of Urology*.

[4] Lin HC, Wu WJ, Ke HL, Huang CH (2006). Bladder leiomyoma presenting as voiding dysfunction: a case report. *Kaohsiung Journal of Medical Sciences*.

[5] Elshebiny YH, Ashebu SD, Hussein HM, El-Naser AMA (2002). Leiomyoma of the urinary bladder: case report. *East African Medical Journal*.

[6] Prate J, Damjanov I, linder J (1996). Disease of the urogenital and reproductive system: female reproductive system. *Anderson’s Pathology*.

[7] Roy MK, Joarder RH, Suruzzaman M (2005). Leiomyoma of the urinary bladder. *Mymensingh Medical Journal*.

[8] Goluboff ET, O’Toole K, Sawczuk IS (1994). Leiomyoma of bladder: report of case and review of literature. *Urology*.

[9] Knoll LD, Segura JW, Scheithauer BW (1986). Leiomyoma of the bladder. *Journal of Urology*.

[10] Fernandez AF, Dehesa TM (1992). Leiomyoma of the urinary bladder floor: diagnosis by transvaginal ultrasound. *Urologia Internationalis*.

